# Algorithm for Image Retrieval Based on Edge Gradient Orientation Statistical Code

**DOI:** 10.1155/2014/705763

**Published:** 2014-04-30

**Authors:** Jiexian Zeng, Yonggang Zhao, Weiye Li, Xiang Fu

**Affiliations:** School of Software, Nanchang Hangkong University, Nanchang, Jiangxi 330063, China

## Abstract

Image edge gradient direction not only contains important information of the shape, but also has a simple, lower complexity characteristic. Considering that the edge gradient direction histograms and edge direction autocorrelogram do not have the rotation invariance, we put forward the image retrieval algorithm which is based on edge gradient orientation statistical code (hereinafter referred to as EGOSC) by sharing the application of the statistics method in the edge direction of the chain code in eight neighborhoods to the statistics of the edge gradient direction. Firstly, we construct the *n*-direction vector and make maximal summation restriction on EGOSC to make sure this algorithm is invariable for rotation effectively. Then, we use Euclidean distance of edge gradient direction entropy to measure shape similarity, so that this method is not sensitive to scaling, color, and illumination change. The experimental results and the algorithm analysis demonstrate that the algorithm can be used for content-based image retrieval and has good retrieval results.

## 1. Introduction

For current content-based image retrieval (CBIR) applications, color, texture, shape, and spatial relationship of image are the most commonly used retrieving features. Generally, shape is the feature linking most closely to the target object, and the object's shape does not vary as the environment changes. Therefore, shape appears to be a consistent feature of an object, and thus a more advantageous retrieving feature, compared to color and texture. Shape representation refers to the technique that processes or calculates objects' shapes, by using a certain method, to form a feature vector. Feature vector should not be sensitive to translation, rotation, and scale changes of the same target object to the largest extent. In general, shape representation methods are divided into two categories [1–3]: edge-based methods and region-based methods.

Among the edge-based feature representation methods, chain code [1] is one of the most important and widely applied representation method. The main idea of edge-based feature is to represent a curve via interconnected segment sequences, formed by a number of the straight segments with specified directions and lengths. A previous study [4] hypothesized that a novel chain code of intersection angles, that is, using a chain of angle-sequence, which is formed by intersection angle between adjacent segments, to represent the curve. Another previous study [5] suggested an improvement on the chain code representation method by hypothesizing a minimal summation statistical direction code (MSSDC), which successfully acquires independence for translation, rotation, scaling invariance, and the starting point. However, the method using chain code to represent the edge shape needs a lot of prior-period image processing works, and the algorithm of extracting chain code is also complicated. In addition, when chain code is applied to represent the images with complex edges, there is a high chance of ambiguity in the trend of the chain code, also restricting the application of chain code in image retrieval.

The gradient direction of image edge not only contains important information of the shape, but also features simpler calculation and lower time complexity. The method representing the shape via edge gradient direction is more operable and feasible, as it requires less prior-period image processing works. The method only requires calculation of edge gradient direction, on a basis of edge detection, but not the other steps, such as dilating image and filling object empty. Therefore, the integration of “edge gradient direction" into image retrieval has attracted strong research interest. From a previous in-depth study on edge gradient direction conducted by Jain and Vailaya [6], edge gradient direction histogram (EGDH) was employed to represent the shape, as well as to facilitate image retrieval; but, EGDH did not possess rotation invariance. Mahmoudi et al. [7] also investigated edge gradient direction and suggested a novel feature vector, namely, edge orientation autocorrelogram (EOAC), to represent the shape. The feature vector was also concerned about the distance relationship between the adjacent edge-pixels, so as to achieve a better reflection on the spatial relationship of the shape, as well as a better result. However, when using this feature vector to represent the rotated image, the result obtained was not ideal. On more studying, [8] introduced a novel method that combined edge gradient direction histogram with edge gradient magnitude histogram, based on salient closed boundary, to represent the shape of the object. Although the method significantly reduced the impact of noise, it neglected key edges. An improved distance coherence vector algorithm was proposed in [9]. The centroid distance of the average coordinates for the biggest connected coherence pixels of image contours is introduced into distance coherence vector as a new feature, which improves the ability to distinguish different shapes. However, the problem that different shapes are with the same descriptor still exists. Besides, the image retrieval algorithm based on distance coherence vector has not achieved ideal effect on retrieving those images polluted by noise; namely, it is with relatively poorer antinoise performance.

In this paper, the statistical method of 8-direction chain code was applied to the statistics for edge gradient direction, so as to propose a novel edge gradient orientation statistical code (EGOSC), which was sequentially used as feature vector to represent the shape. By constructing *n*-direction vector and setting max-sum constraint, the rotation invariance of EGOSC was ensured. Afterwards, EGOSC was employed for shape-based image retrieval, for proposing a shape match method called Euclidean distance of direction entropy accordingly. Last but not least, Euclidean distance of edge gradient direction entropy was applied, to measure shape similarity, and thus making this method not sensitive to the variation in scale, color and illumination.

## 2. The Algorithm Description of EGOSC

### 2.1. Edge Detection

Sobel operator is an edge detection operator which is not sensitive to noise and is accurate in edge positioning. Therefore, Sobel operator was used for edge detection in this paper.

### 2.2. The Computation of Edge Pixel Amplitude and Edge Pixel Gradient Orientation Angle

Here, amplitude of edge pixels is defined as

(1) $$\begin{array}{c} M\left(x,y\right)=\sqrt{{\left(\frac{\partial f\left(x,y\right)}{\partial x}\right)}^{2}+{\left(\frac{\partial f\left(x,y\right)}{\partial y}\right)}^{2}} \end{array}$$

and gradient orientation angle of edge pixels is defined as

(2) $$\begin{array}{c} A\left(x,y\right)=\text{ta}{\text{n}}^{-1}\left(\frac{\partial f\left(x,y\right)/\partial y}{\partial f\left(x,y\right)/\partial x}\right), \end{array}$$

where*f*(*x*, *y*)/∂*y* = *f*(*x*, *y* + 1) − *f*(*x*, *y* − 1) and ∂*f*(*x*, *y*)/∂*x* = *f*(*x* + 1, *y*) − *f*(*x* − 1, *y*). ∂*f*(*x*, *y*)/∂*y* and ∂*f*(*x*, *y*)/∂*x* refer to the vertical and horizontal gradient of image, respectively. *f*(*x*, *y*) refers to image grayscale.

### 2.3. The Determination of the Salient Edges

Thresholding is the method that does not only detect the salient edges of the image, but also filters off the noise in the image. In this paper, the salient edges of the image were extracted by comparing image gradient amplitude with a given threshold *T*; that is, the edge pixels where *M*(*x*, *y*) > *T* were selected as salient edge pixels, and other edge pixels were removed. In general, threshold *T* is automatically searched by the program using threshold iteration method.

### 2.4. The Establishment of EGOSC

As shown in (2), the limit of variation for edge gradient orientation is [−*π*/2, *π*/2]. For convenient representation and calculation, the limit of variation for edge gradient orientation is transformed to [0, *π*].

In order to illustrate and represent the overall shape and the local details of the edge, the limit of variation for edge gradient orientation is equally divided into *n* intervals; that is, [0, *π*] is equally divided into *n* direction intervals with a unit of *π*/*n*. The limit of variation for the first direction interval is expressed as [0, *π*/*n*), the limit of variation for the *i*th direction interval is expressed as [(*i* − 1)*π*/*n*, *iπ*/*n*), and the limit of variation for the *n*th direction interval is expressed as [(*n* − 1)*π*/*n*, *π*], where *i* represents the *i*th interval, and *n* represents the number of equally divided intervals within the limit of variation for edge gradient orientation. The value of *n* largely depends on complexity of the edge. For edge with higher complexity, *n* should be given a higher value, so as to fully illustrate and represent the local details of the edge; for the edge with moderate complexity, *n* = 18 is good enough to represent a clear and detailed edge shape.

After edge gradient orientation is quantized to *n* direction intervals, the numbers of edge pixels located at each direction interval are counted statistically, before the formation of a row vector. If *X* is used to denote the row vector, then

(3) $$\begin{array}{c} X=\left(\begin{array}{cccccc} x_{1} & x_{2} & \cdots & x_{i} & \cdots & x_{n} \end{array}\right), \end{array}$$

where *x*_*i*_ | *i* = 1,2,…, *n* refers to the number of edge pixels located at the *i*th direction interval.

Equation (3) is herein defined as EGOSC.

### 2.5. The Construction of *n* Direction Vector

In order to simplify the computation, the angle range of each interval is represented by an interval; that is, *n* intervals are represented by *n* integers, and *n* direction vector is defined as follows:

(4) $$\begin{array}{c} D={\left[\begin{array}{cccccc} 1 & 2 & \cdots & i & \cdots & n \end{array}\right]}^{T},\;i=1,2,\ldots ,n, \end{array}$$

where 1 represents [0, *π*/*n*) direction interval, 2 represents [*π*/*n*, 2*π*/*n*] direction interval, *i* represents [(*i* − 1)*π*/*n*, *iπ*/*n*] direction interval, and *n* represents [(*n* − 1)*π*/*n*, *π*] direction interval.

### 2.6. The Rotation of EGOSC

After the rotation of EGOSC, *n* rotated EGOSCs are obtained. The initial EGOSC of the object contour is set as $X_{1}=\left(\begin{array}{cccccc} x_{1} & x_{2} & \cdots & x_{i} & \cdots & x_{n} \end{array}\right)$, and if the image is rotated in a clockwise direction, the following rule would be demonstrated:

(5) $$\begin{array}{c} X_{i}=\left\{\left(\begin{array}{ccc} x_{(i+1)⊕n} & x_{(i+2)⊕n\;\cdots } & x_{(i+n)⊕n}\;|\;i=0,1,\ldots ,n \end{array}\right)\right\}, \end{array}$$

where ⊕*n* denotes modulo-*n* operation, since the variation cycle of EGOSC is equal to *n*.

**Figure 1:**
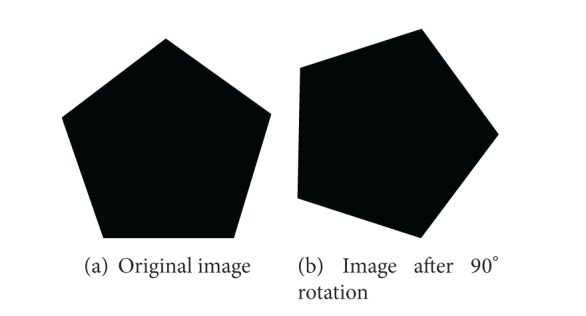
Image of pentagon.

As shown in Figure 1, Figure 1(b) is the image obtained by rotating Figure 1(a) in a clockwise direction by 90 degrees. When given *n* = 18, EGOSC of Figure 1(a) is as follows:

(6) $$\begin{array}{c} X_{1}=\left(150\;87\;26\;22\;21\;121\;1\;1\;3\;430\;3\;1\;117\;23\;15\;23\;96\;1\right), \end{array}$$

and EGOSC of Figure 1(b) is as follows:

(7) $$\begin{array}{c} X_{10}=\left(430\;3\;1\;117\;23\;15\;23\;96\;1\;150\;87\;26\;22\;21\;121\;1\;1\;3\right). \end{array}$$

#### 2.6.1. The Max-Sum Constraint of EGOSC

The max-sum constraint of EGOSC is defined as follows:

(8) $$\begin{array}{c} D_{\max}=\max\left\{X_{i}\cdot D\;|\;i=1,2,\ldots ,n\right\}, \end{array}$$

where {*X*_*i*_ | *i* = 1,2,…, *n*} represents *n* EGOSCs that are obtained from *n* rotations, and *D* represents *n* direction vector which is a column vector. The purpose of computing the max-sum constraint of EGOSC is to select one of *n* EGOSCs obtained from *n* rotations as the final shape-representing code.

As shown in (5), EGOSC depicts a systematic change during the rotation of images. Therefore, the use of (8) to set max-sum constraint on EGOSC is actually to fix the shape to the same reference angle as *n* directions, so as to solve the problem of the rotation sensitivity. Only the orientation statistical code satisfying the equation of max-sum constraint is eligible to be applied as the sole index corresponding to the contour.

If there is more than one EGOSC satisfying (8), the first EGOSC encountered during the clockwise rotation is referred to as the final representation. For example, when *n* = 18, EGOSC for an image after 18 clockwise rotations is *X*_*i*_ = [*X*_1_ *X*_2_ *L* *X*_18_]^*T*^, which multiplies the direction vector *D* to obtain *X*_*i*_ · *D* = [150 150 134 128 37 66 58 79 16 89 145137 44 56 34 57 90 101], to obtain two maximum values of 150 that satisfy (8); thus, the *X*_*i*_ corresponding to the first 150 should be selected as the final representation. EGOSC is written as a row vector.

**Figure 2:**
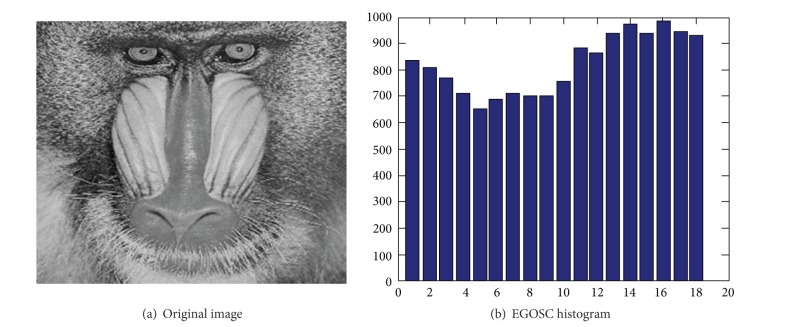
Image of baboon and its EGOSC histogram.

**Figure 3:**
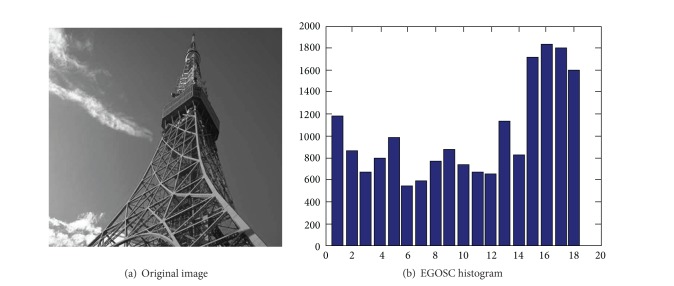
Image of an iron pagoda and its EGOSC histogram.

Figures 2(a) and 2(b) show a baboon image and its EGOSC histogram (the limit of variation for edge gradient orientation is equally divided by *n*, where *n* = 18), respectively. Figures 3(a) and 3(b) show an iron pagoda image and its EGOSC histogram (the limit of variation for edge gradient orientation is equally divided by *n*, where *n* = 18). In the histogram, the horizontal axis represents 18 directions, and the vertical axis represents the number of the edge pixels in the 18 directions. In this paper, this row vector is employed as the feature vector to represent the shape of the images, as well as the retrieval feature used in content-based image retrieval (CBIR).

## 3. Similarity Measurement: Euclidean Distance of Edge Gradient Directional Entropy

EGOSC is a vector statistically reflecting the features of image. Any variation in the scale and illumination of the image will cause variation in number of image edge pixels, and thus resulting in changes of EGOSC. In order to eliminate such influence, entropy of edge gradient orientation code (hereinafter called direction entropy) is employed in this paper, for normalizing this feature vector. According to the idea of a previous study [10], EGOSC could be considered as a set of strings, and the information entropy could be used to measure the distribution pattern of shape in various directions. Another previous study [5] verified that similar image shared similar direction entropy, constituting a feature for image retrieval. On more studying, [11] directly sets the entropy of smooth points between contour corners as a feature for image retrieval.

According to the theory of Shannon information entropy, the edge gradient direction entropy is thus defined as follows.

If *X* = (*x*_1_ *x*_2_ *K* *x*_*i*_ ⋯ *x*_*n*_) is the edge gradient direction code, and the occurrence probability of the pixels constituting the edge contour in *n* directional intervals is

(9) $$\begin{array}{c} p_{i}=\frac{x_{i}}{\sum_{i=1}^{n}x_{i}}. \end{array}$$

Then, the edge gradient direction entropy should be

(10) $$\begin{array}{c} E_{D}=\overset{n}{\underset{i=1}{\sum }}e_{i}, \end{array}$$

where *e*_*i*_ = −*p*_*i*_log_2_(*p*_*i*_).

If the information of a particular shape is viewed as an information source, then −log_2_(*p*_*i*_) represents the amount of shape information distribution in each direction, while *E*_*D*_ reflects the average amount of shape information distribution in *n* directions. To a certain extent, *E*_*D*_ reflects the characteristic of the shape.

A major issue of shape matching is the distance measurement among feature vectors. Many similarity measurement methods have been reported [12, 13]. The most common methods to measure the similarity of space distance are Euclidean distance, Mahalanobis distance, Minkowski distance, city-block distance, and so forth. Based on well-defined direction entropy, *e*_*i*_ is used as the feature vector for retrieval in this paper, and Euclidean distance of the direction entropy is defined as follows:

(11) $$\begin{array}{c} S\left(Q,I\right)=\overset{n}{\underset{i=1}{\sum }}{\left[{\left(e_{Qi}-e_{Ii}\right)}^{2}\right]}^{1/2}, \end{array}$$

where *e*_*Qi*_ refers to the direction entropy of the queried image in the *i*th direction, *e*_*Ii*_  (*I* = 1,2,…, *m*) refers to the direction entropy of each image in the image library in the *i*th direction, and *m* refers to the number of images in the image library.

*S*(*Q*, *I*) measures the degree of shape similarity between queried image *Q* and image *I* in image library, and smaller value of *S*(*Q*, *I*) means image *Q* and image *I* are more similar. In this paper, (11) is used to calculate image similarity, *S*(*Q*, *I*), before sequencing the values in an ascending order (i.e., from low to high). Afterwards, the previous *r* images are returned according to the presettings.

## 4. The Invariance Analysis of the Feature Vector

The matching algorithm for measuring the similarity during image retrieval should be as close to human eye judgment as possible. In human's eyes, the similarity between two images of one same objective is distinguished by the five variant factors, that is, translation, rotation, scale, color, and illumination [7]. Therefore, a good image retrieval system should maintain the invariance for the above 5 features. In this part of the paper, the performance of EGOSC for maintaining the invariance is discussed. EGOSC possesses translation invariance because translation of an image would not affect the amplitude and direction of the edge.

### 4.1. The Analysis on Scaling Invariance

**Figure 4:**
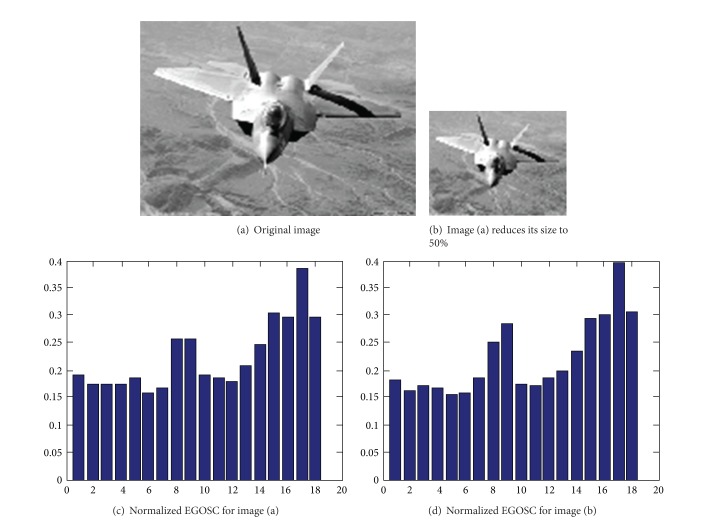
EGOSC histograms for images with varied scaling.

As the size of the image determines the number of edge pixels, scaling variation could readily affect EGOSC. However, EGOSC will vary proportionally to the scaling variation. As a result, the influence of image scaling variation could be eliminated by the normalization. As shown in (10), the edge gradient direction entropy reflects the probability distribution of the pixels composing the edge contour in *n* directional intervals. For the image with changed scale, the use of edge gradient direction entropy could lead to the normalization of EGOSC.  Figure 4 shows a comparison between EGOSC histograms before and after scaling variation. From Figures 4(c) and 4(d), it shows that EGOSC after normalized by edge gradient direction entropy possesses good invariance against the scaling variation.

### 4.2. The Analysis on Color and Illumination Invariance

**Figure 5:**
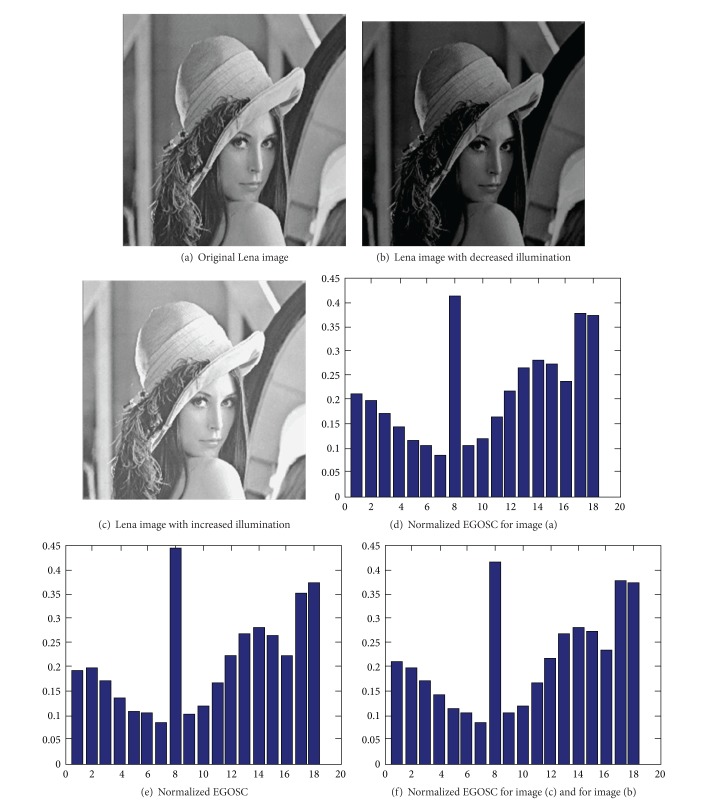
EGOSC for Lena image with varied illumination.

The variation in the color and illumination of image could result in a change of image pixel intensity. For example, enhancing the illumination of an image can lead to an increase in pixel gray value of the image. The use of Sobel operator for edge detection will affect the amplitude of image gradient *M*(*x*, *y*), and the number of pixels largely depends on both fixed threshold *T* and gradient amplitude *M*(*x*, *y*). Therefore, the variation in illumination and color will influence the total number of edge pixels, so as to alter EGOSC. However, the variation in color and illumination of the image do not affect the edge direction of the image. It is because the edge is constructed within the boundary regions and the relative positions of these boundary regions remain invariant. In other words, the variation in color and illumination of the image only affects the number of edge pixels, but not the edge direction of the image. Reflecting on EGOSC, the variation of EGOSC is proportional to the variation in illumination. As a result, the influence of illumination variation could be readily eliminated by the normalization of direction entropy.  Figure 5 shows EGOSCs for images with varied illumination.

As shown in Figure 5, the EGOSCs for the images with varied illumination are not much differed after being normalized by direction entropy generally, indicating that the method possesses good illumination invariance.

### 4.3. The Analysis on Rotation Invariance

The limit of variation for edge gradient orientation is equally divided into *n* parts; that is, [0, *π*] is equally divided into *n* direction intervals with a unit of *π*/*n*.

**Figure 6:**
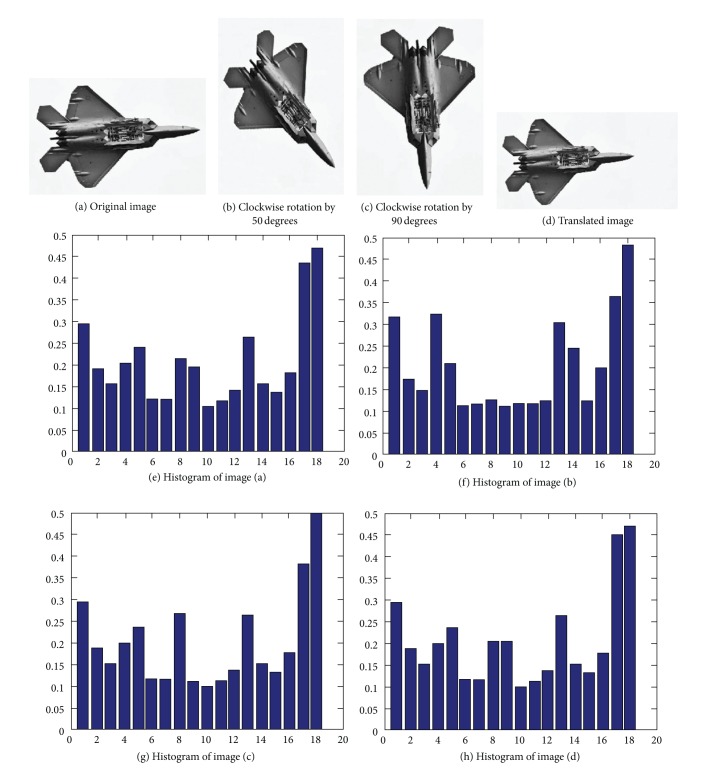
The effects of image rotation and translation on EGOSC.

The algorithm in this paper is to quantize the orientation of each edge pixel into *n* direction intervals with a unit of *π*/*n* and to set the max-sum constraint by (8). By doing this, each image is rotated to the same perspective so that a better rotation invariance is achieved when the rotation angle is larger (greater than the quantized angle). The experiment also proves the effectiveness of this algorithm.  Figure 6(a) is the top view of a F22 fighter, Figure 6(b) is the rotated image of Figure 6(a) in a clockwise direction by 50 degrees, Figure 6(c) is the rotated image of Figure 6(a) in a clockwise direction by 90 degrees, and Figure 6(d) is the translated image of Figure 6(a). Figures 6(e), 6(f), 6(g), and 6(h) are the corresponding EGOSC histograms to Figures 6(a), 6(b), 6(c), and 6(d), respectively. As shown in Figure 6, EGOSCs of translated or rotated images are not much different from that of the original image generally, indicating that the method possesses good rotation invariance.

## 5. The Experimental Results and Analysis

### 5.1. The Evaluation Mechanism of the Algorithm

Precision rate and recall rate are two criteria used to evaluate the image-retrieving performance of an algorithm. Herein, precision rate refers to the ratio of the number of the target images in the retrieval result sequence to the total number of images in the sequence, while recall rate refers to the ratio of the number of the target images in the retrieval result sequence to that in the entire image library. For different image retrieval algorithms, those with higher precision rates under same recall rate are more favorable as they present better retrieval results. In this paper, precision rate was used to measure the performance of the proposed algorithm, in which, the precision rate is calculated as follows: *P*_*G*_ = *l*/*G*, where *G* refers to the total number of the output images from the image retrieval system, while *l* refers to the number of images in the image class to which the queried image *Q* belongs. In addition, for the purpose of examining the performance of this algorithm, the algorithm was further compared with the conventional edge direction histogram (EDH) algorithm [6] and the edge orientation autocorrelogram (EOAC) algorithm [7].

### 5.2. The Experimental Results and Discussion

In order to examine the ability to retrieve images with complex edges, 5 classes of images with complex edges from the Internet were selected as image library in this paper, including fighters, flowers, mountains, toys, and fruits. Each class contained 50 images, and a total of 250 images were selected. From each class, several images were randomly selected to perform various transformations, such as rotation and varying the scale, before returning to the image library. Afterwards, another 5 images were randomly selected from each class as the queried images. *P*_10_ and *P*_20_ of each image were calculated. Last but not least, the average precision rates $\bar{P_{10}}$ and $\bar{P_{20}}$ for each class were also calculated, respectively.

Table 1 shows the average precision rates of the three algorithms for 5 classes of images. $\bar{P_{10}}$ represents the average precision rate of the first 10 retrieval results returned, while $\bar{P_{20}}$ represents the average precision of the first 20 retrieval results returned. From the data in Table 1, the average precision rates of this proposed algorithm were relatively higher compared to others.

**Figure 7:**
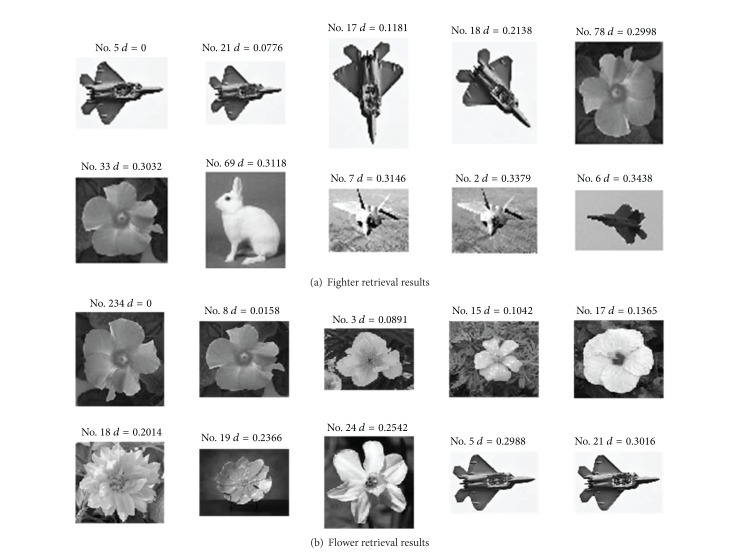
Retrieval results for EGOSC.

**Figure 8:**
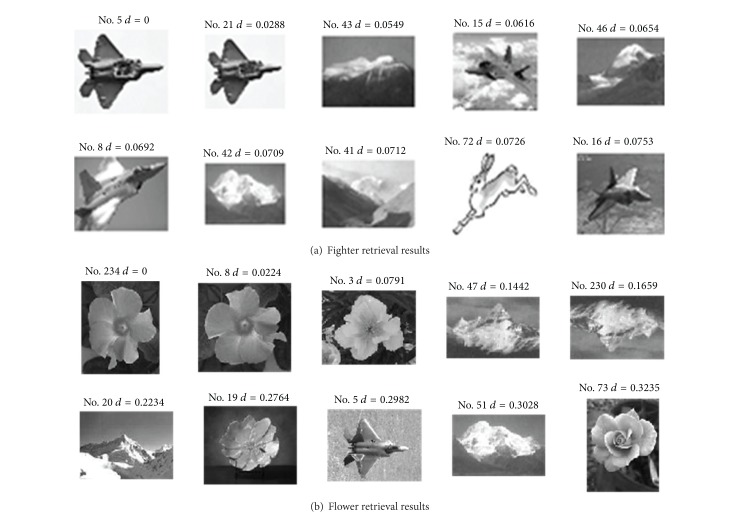
Retrieval results for EDH.

**Figure 9:**
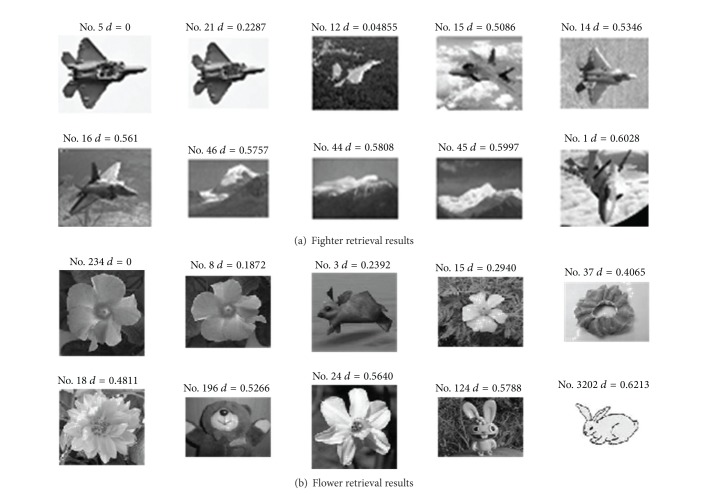
Retrieval results for EOAC.

Figure 7 shows the retrieval results of fighters (Figure 7(a)) and flowers (Figure 7(b)) in the image library acquired by the proposed algorithm. The first image is the queried image, which is also an image indeed existing in the library, while the other 9 images are the images with the highest similarity to the queried image. As the control, Figures 8 and 9 show the retrieval results acquired by EDH and EOAC, respectively. As shown in Figures 8 and 9, EDH and EOAC acquired poor retrieval results if the images were rotated. In contrast, the proposed algorithm could readily resolve this problem.

The experimental results showed that the proposed EGOSC could be used as a mean of CBIR. The construction of max-sum constraint on the 18 directional vectors for EGOSC ensured excellent rotation invariance to the proposed algorithm. Also, the measurement of shape similarity by defined Euclidean distance of direction entropy ensured scale and illumination invariance to the proposed algorithm. Among the three algorithms which take edge direction as feature vector (EGOSC, EDH, and EOAC), the proposed algorithm (EGOSC) acquired relatively better retrieval results. In addition, the proposed algorithm could acquire good retrieval results when dealing with the image libraries with definite objective and single background.

The proposed algorithm is an easily feasible method with a low time-complexity, and it could readily solve the problems raised by the image transformation, such as rotation, translation, and scale variation. However, EGOSC only presents the characteristics on the statistical aspect, but not those regarding spatial distribution. Therefore, images with different shapes may share the same EGOSC.

## 6. Conclusions

In this paper, a novel algorithm for image retrieval based on EGOSC was proposed. EGOSC was constructed by statistically computing the gradient direction based on edge detection. The construction of max-sum constraint on the 18 directional vectors for EGOSC ensured excellent rotation invariance to the proposed algorithm. Also, the measurement of shape similarity by defined gradient direction ensured scale and illumination invariance to the proposed algorithm. From the results of abundant experiments, the proposed algorithm could acquire excellent retrieval results, and thus could be used as a potential mean of CBIR.

## Figures and Tables

**Table 1:** Comparison of the average precision rates between proposed algorithm and others (EOAC, EDH).

Target class	$\bar{P_{10}}$%			$\bar{P_{20}}$%		
	Proposed	EOAC	EDH	Proposed	EOAC	EDH
Flower	70	50	52.5	68	58.7	61.3
Fruit	71	56.7	65	53	56.7	50
Mountain	60	54	53.5	45	48	49
Toy	48	50	32	35	47	31
Fighter	37	35	25	32	30	19
